# A Guide to the Practical Examination of Urine for the Use of Physicians and Students

**Published:** 1896-02

**Authors:** 


A GUIDE TO THE PRACTICAL EXAMINATION OF URINE for the use of Physicians and Students, by James Tyson, M. D., Professor of Clinical Medicine in the University of Pennsylvania, and Physician to the Hospital of the University, etc., etc. Ninth Edition. Revised and corrected, with a colored plate and forty-eight wood cuts. Price, cloth, $1.25. P. Blakiston, Son & Co., Publishers, 1012 Walnut St., Philadelphia. 1895.

This book is of small and convenient size for use as a practical guide in the laboratory or office. It contains 276 pages, and is filled with practical instruction for the thorough chemical and microscopic examination of urine in health and disease. A French translation of this very successful book has just appeared in Paris under the editorship of Drs. Gautrelet and A. S. Clarke, and published by the Societe d'Editions Scientifiques. H.

P. BLACKISTON, SON & Co., of Philadelphia, announce a book on "Appendicitis," by John B. Deaver, M. D., Assistant Professor of Applied Anatomy, University of Pennsylvania, Assistant Surgeon to the German Hospital, etc. The book will be arrangdd in a practical and systematic manner. The history, etiology, symptoms, diagnosis, operative treatment, prognosis, and complications of this disease will be given in the order named. It will contain about forty illustrations of methods of procedure in operating, and typical pathological conditions of the appendix, the latter being printed in colors.

